# A rare case report on diabetes insipidus following living donor liver transplantation in patient with alcoholic liver disease

**DOI:** 10.6026/973206300214939

**Published:** 2025-12-15

**Authors:** Vivekanandan Shanmugam, B. Aanand Shankar

**Affiliations:** 1 Department of liver transplantation, RPS Hospitals, Chennai, Tamil Nadu, India; 2 Department of liver transplantation, Chennai Liver Foundation, Chennai, Tamil Nadu, India; 3 Department of anaesthesiology, RPS Hospitals, Chennai, Tamil Nadu, India; 4 Department of anaesthesiology, SIMS Hospital, Vadapalani, Tamil Nadu, India

**Keywords:** Living donor liver transplantation, polyuria, nephrogenic diabetes insipidus, anti-diuretic hormone, desmopressin, case report

## Abstract

A rare occurrence of nephrogenic diabetes insipidus (DI) following liver transplantation despite elevated plasma antidiuretic hormone levels and normal brain imaging findings is of interest. On the sixth day post-transplantation, the patient developed polyuria, a hallmark symptom of DI, prompts a comprehensive differential diagnosis. Desmopressin therapy was initiated, leading to rapid symptom resolution and full recovery. This case underscores the diagnostic challenges of DI in liver transplant recipients, particularly in differentiating between central and nephrogenic forms. Conventional diagnostic tests for DI, like water deprivation, are not preferred in patients receiving liver transplantation. Therefore, clinical assessment, biochemical markers, and imaging play a crucial role in diagnosis. The patient was successfully managed with desmopressin, thus emphasizing the importance of early recognition of symptoms and prompt initiation of treatment to prevent complications in liver transplantation patients to ensure optimal outcomes. Thus, we show the potential for rare post-transplant complications and the need for individualized diagnostic and therapeutic strategies.

## Background:

Diabetes insipidus (DI) is a debilitating disorder with a prevalence of 1 in 25,000. DI can present at any age with a similar prevalence in males and females. Diagnosis of DI is established by a two-stage dehydration test and a vasopressin test that differentially identifies between neurohormonal/central DI and nephrogenic DI. The treatment of DI depends upon its cause. The water deficit and electrolyte balance should be corrected and stabilized along with relieving excessive thirst and reducing excessive urine production. For symptom management of DI, long-acting vasopressin analog (desmopressin) is recommended via the intranasal or oral route [1, 2]. With the resolution of clinical symptoms, plasma osmolarity returns to normal, and the sodium in the serum normalizes [3]. Post-operative DI is a rare complication following liver transplantation. Understanding the occurrence and clinical characteristics of DI in this setting is crucial, as only a few cases have been reported. Early diagnosis and treatment of DI are critical. Dehydration during the recovery period after liver transplantation can lead to electrolyte imbalances and life-threatening complications [4, 5]. This report describes a 57-year-old male with alcohol-related decompensated chronic liver disease who underwent living donor liver transplantation (LDLT) and subsequently developed post-operative DI. Therefore, it is of interest to emphasize the clinical course of post-operative DI, the importance of early symptom identification, prompt diagnosis, and the implementation of standard treatment in post-transplant patients.

## Case report:

A 57-years-old male with known history of decompensated chronic liver disease and portal hypertension secondary to alcohol use disorder was hospitalized for LDLT. His body mass index (BMI) was 25.1 kg/m^2^. The patient had been experiencing symptoms of decompensated liver disease for 8 months, manifested by an incidence of upper gastrointestinal bleed that required endoscopic variceal ligation (EVL). The patient had experienced a seizure episode one year ago, but no treatment was initiated, and no further seizures were reported. The patient also had a history of hypertension, diagnosed two years ago, and hypothyroidism for the past three years. He was taking oral thyroxine (50 mcg, once daily) to manage hypothyroidism. The patient has had vitiligo since childhood and was receiving appropriate medical management as per the dermatologist's advice, though specific details of the treatment are not available. He had undergone bilateral cataract surgery three years ago and dental implantation a month ago (Figure 1 - see PDF). Upon admission, his vital parameters were normal (temperature: 97.4 °F, heart rate: 94 beats per minute, blood pressure:140/80 mmHg). Cardiovascular assessment revealed normal heart sounds (S1, S2), while respiratory findings indicated bilateral air entry (BAE). Neurological examination was unremarkable, with non-focal neurological deficits noted. The abdomen was soft with minimal distension, and the patient showed signs of jaundice. The patient's weight was recorded at 69 kg, showing an increase from 58 kg upon initial admission. Pre-operative laboratory findings showed abnormalities in the coagulation profile, with an increase in factor VIII and a decrease in antithrombin levels, indicating impaired liver functions (Table 1 - see PDF). The autoimmune panel was positive for both antinuclear antibodies (ANA) and antineutrophil cytoplasmic antibodies (ANCA). The patient had a model for end-stage liver disease (MELD) score of 23. Pre-operative thyroid gland function was normal (preoperative thyroid stimulating hormone level [TSH]: 0.975 mIU/L [normal range: 0.4 to 4.5 mIU/L]). The anesthetic team adopted the standard protocol of LDLT surgery as summarized in Table 2 (see PDF). The patient underwent LDLT with a graft from his 19-year-old daughter, who had no medical history of liver or other disorders. The surgery was performed following a standard orthotopic right lobe implantation without complete inferior vena cava clamping. The graft-to-recipient mass ratio was 1.13 and the operative duration was 7.5 h. Due to intra-operative bleeding, the patient received two units each of packed red cells and fresh-frozen plasma, five units of cryoprecipitate, and tranexamic acid (4 gm). The intraoperative hemodynamic status remained stable. The patient was successfully extubated on the same day following the procedure. To prevent post-operative infections, a comprehensive antibiotic regimen was initiated, including intravenous piperacillin-tazobactam (4.5 g), teicoplanin (400 mg), and fluconazole (200 mg). Methylprednisolone (625 mg) was administered to suppress the immune response and prevent rejection. Supportive measures, such as albumin (20%, four units) administration, electrolyte replacement (magnesium, bicarbonate, and calcium), and diuretic therapy (furosemide 20 mg), were employed to maintain fluid and electrolyte balance. Total fluid input was 4150 mL, and total output was 2000 mL, indicating close monitoring of fluid balance throughout the procedure. Post-operative laboratory evaluations are presented in Table 3 (see PDF). A brain magnetic resonance imaging (MRI) scan was conducted to rule out central DI due to any damage to the hypothalamus or pituitary. No abnormalities were detected, and no areas of restricted diffusion were observed. Age-related involutional changes were noted, including prominence of the cortical sulci, cerebellar folia, ventricular system, and basal cisternal spaces. Mild periventricular hyperintense signals, consistent with small vessel ischemic disease, were observed. A chronic lacunar infarct with gliosis was identified in the head of the right caudate nucleus, along with associated dilation of the frontal horn and anterior body of the right lateral ventricle. Additionally, a few chronic lacunar infarcts appeared hyperintense on T2-weighted and fluid-attenuated inversion recovery (FLAIR) sequences in the bilateral frontal, periventricular, and subcortical white matter. No signs of acute infarction, hemorrhage, or mass lesions were detected. The rest of the brain parenchyma showed normal signal intensities without any focal lesions. The brain stem and cerebellum appeared normal. Overall, the findings were consistent with age-related involutional changes, mild small vessel ischemic disease (Fazeka 1), and a few chronic lacunar infarcts. The pituitary was normal, and no other significant abnormalities were noted. The patient was started on desmopressin nasal spray (20 mcg) twice daily and transitioned to oral desmopressin tablets 0.1 mg twice daily after two days. The treatment was stopped after five days on day POD 10. The patient was discharged on POD 17. He responded well to the treatment and had no complaints.

## Follow-up and outcomes:

Post-discharge, the patient remained asymptomatic with no recurrence of polyuria or polydipsia and reported a normal daily routine. Importantly, there was no evidence of DI. Physical examination revealed no abnormalities. The patient underwent routine follow-up after liver transplantation, and routine lab work was within normal ranges. The patient reported complete remission of his initial polyuria and polydipsia. He expressed satisfaction with his overall health and quality of life and has fully resumed his daily activities. On postoperative day (POD) 6, the patient started producing large quantities of urine (urine output: 7-8 L/day without diuretics) with high antidiuretic hormone (ADH) levels (73 pg/mL) raising a suspicion of nephrogenic DI (urine osmolality: 405 mOsm/kg [normal], serum osmolality: 255 mOsm/kg [low], urine specific gravity: 1.015, serum sodium: 135 [normal] mEq/L, and serum and urine glucose were within normal limits. His blood pressure was 130/60 mmHg, and heart rate was 98 beats per minute.

## Discussion:

In this case report, we describe the rare incidence of nephrogenic DI in a patient six days after undergoing LDLT. The patient developed polyuria and high levels of plasma ADH. The MRI of the brain did not reveal any significant structural abnormalities that could explain the etiology of DI. The patient was immediately initiated on desmopressin therapy, which was continued for five days. Desmopressin therapy resulted in symptom resolution and complete recovery. The distinctive diagnostic features of DI include polydipsia and polyuria (urine output in adults: >40 mL/kg/day and urine output in children: >100 mL/kg/day) accompanied by excretion of large volumes of dilute urine (urine osmolality: <300 mOsm/L). This can either occur due to either insufficient production of ADH, or inability of the kidneys to respond to ADH. As a result, the kidneys are unable to concentrate urine, leading to the passage of large amounts of dilute urine [2]. In the present case, the patient started producing large quantities of urine on POD 6 (urine output: 7-8 L/day) without diuretics. The urine osmolarity was within normal range, but the serum osmolarity was low. Serum sodium and glucose, and urine glucose were also within normal range. His blood pressure was 130/60 mmHg, and his heart rate was 98 beats per minute. The probable cause of polyuria was suspected to be DI. DI can be classified into central DI (caused by insufficient ADH production), nephrogenic DI (caused by non-responsiveness of the kidneys to ADH), and gestational DI [6]. Owing to the higher-than-normal levels of ADH in the present case, the underlying etiology of DI was suspected to be nephrogenic. In nephrogenic DI, the plasma ADH levels are high, to counter the renal resistance to its action. In central DI, plasma ADH levels remain relatively low despite elevated plasma osmolality [7]. The main causes of nephrogenic DI reported in literature are genetic, hematological disorders, medications, electrolyte abnormalities (hypokalemia and hypercalcemia), renal injury, and chronic kidney disease [8]. The differential diagnosis of nephrogenic DI can be challenging, considering the substantial variations in urine output and osmolality [1]. Other diagnostic approaches that can be considered include the water deprivation test and the desmopressin challenge [9]. The principle of the water deprivation test is to withhold all fluids from the patient to determine the subject's dehydration status. Due to the risk of pronounced dehydration and hypovolemia, such tests are not considered suitable for liver transplantation patients. The Enhanced Recovery after Surgery (ERAS) guidelines strongly recommend a goal-directed fluid therapy for patients undergoing liver transplantation, based on non-invasive monitoring of central blood pressure and cardiac output. Both excessive and restrictive fluid therapy poses risks. Excessive fluid infusion can lead to severe complications like interstitial edema, lung injury, and bleeding, while restrictive fluid therapy can increase the risk of renal failure. Furthermore, low portal blood flow can compromise graft liver function [10]. The MRI of the brain is another useful strategy to identify any structural abnormalities that may be causing DI. In the present study, the brain MRI did not reveal any evidence of damage to the hypothalamus or pituitary, and no abnormality was detected. The high urine osmolality and high ADH levels were indicative of a renal concentrating defect, confirming the diagnosis of DI. While the exact mechanism underlying the development of nephrogenic DI post-liver transplantation remains unclear, potential factors may include drug-induced nephrotoxicity, electrolyte imbalances, or direct renal injury. Our case report adds to the limited literature on nephrogenic DI following liver transplantation. Only two cases of central DI have been reported in liver transplant recipients (Table 4 - see PDF). In the first report, a 56-year-old female with non-alcoholic steatohepatitis developed central DI after LDLT due to pituitary apoplexy and was treated conservatively. Surgery was not required [4]. In the second case, a 77-year-old male diagnosed with hepatitis B-related chronic liver disease with hepatocellular carcinoma developed idiopathic central DI after liver transplantation. Desmopressin treatment of polyuria and central DI resulted in prompt symptom relief in the second case [5]. Both cases were managed successfully, highlighting the importance of early diagnosis and treatment of DI in liver transplant recipients. The present case presents similar features of DI to the previously described subjects. Polyuria appeared after liver transplantation. However, there were some striking differences. Our patient did not have any history of head trauma or damage to brain pre- or perioperatively. The brain MRI did not show any abnormality and a normal appearance in T1-weighted images. The results of serum biochemistry and urine tests were compatible with nephrogenic DI. This patient had no discernible cause but showed high levels of ADH; therefore, DI was thought to be idiopathic and nephrogenic.

After diagnosis, the treatment of DI entails the ADH analog desmopressin administration as an intranasal spray, tablet, or sublingual melting formulation. Desmopressin is delivered two to three times daily to alleviate symptoms of polyuria and polydipsia. Desmopressin is the mainstay of treatment for patients with DI, due to its long half- life, selectivity for arginine vasopressin receptor 2 (AVPR2) and the availability of various pharmaceutical formulations [6, 11]. The dose and regimen should be optimized for patients depending on their clinical presentation. In the present case, the patient was immediately initiated on a desmopressin nasal spray owing to superior consistency of absorption and efficacy, and later he was switched to the convenient regimen of oral tablets twice daily. The maximum dose of desmopressin required is 0.2 mg orally, 120 mcg sublingually or 10 mcg (one nasal spray) two or three times daily. These results in plasma desmopressin levels higher than those required for maximum antidiuresis but these doses decrease the need for more frequent treatment [3]. This case report highlights the diagnostic challenges and management strategies for DI following liver transplantation. Establishing the correct diagnosis of polyuria is crucial in this setting. Our primary aim is to raise awareness among transplant surgeons and intensivists about the occurrence of idiopathic DI in patients undergoing liver transplantation. It is important to note that the findings of this report may not be applicable to all patients and do not necessarily imply a cause-and-effect relationship. Additionally, the retrospective nature of data collection in this study may have introduced recall bias. In the present case, fluid restriction could not be done anticipating the risk of reducing portal flow that could have impaired the graft liver perfusion and function, and worsened the patient's condition. A urine specific gravity calculation and an MRI scan seemed more relevant and safe assessments. In addition to no visible alterations in the MRI scan, the prompt response to desmopressin treatment confirmed the initial diagnosis.

However, further research is crucial to elucidate the underlying mechanisms and risk factors leading to the development of DI post-transplantation. Despite these limitations, the present case emphasizes the importance of early recognition of DI symptoms, close monitoring of patients post-operatively, maintaining optimal fluid balance, and prompt initiation of treatment. This approach is essential to avoid complications and ensure optimal patient outcomes in this setting.

## Conclusion:

The differential diagnosis of DI is challenging in patients immediately after transplantation. In our case, the patient responded well to desmopressin therapy and the treatment was stopped on the fifth day, thus emphasizing the importance of timely intervention to manage transient DI post-transplantation. To improve clinical outcomes, it is imperative for transplant physicians, intensivists, and nephrologists to be aware of the rare complication of DI and its successful management in patients undergoing liver transplantation.

## Author contribution:

All authors performed the surgery and postoperative management of the patient and contributed equally to the case discussion and manuscript writing. All authors have read and approved the final manuscript.

## Funding:

None of the authors received any funding for the study.

## Human/Animal Rights:

All procedures followed were in accordance with the ethical standards of the responsible committee on human experimentation (institutional and national) and with the Helsinki Declaration of 1975, as revised in 2008(5).

## Informed consent:

Informed consent was obtained from the patient included in the study.
